# Metabolic Reprogramming Induces Macrophage Polarization in the Tumor Microenvironment

**DOI:** 10.3389/fimmu.2022.840029

**Published:** 2022-07-07

**Authors:** Shilin Wang, Guohong Liu, Yirong Li, Yunbao Pan

**Affiliations:** ^1^ Department of Laboratory Medicine, Zhongnan Hospital of Wuhan University, Wuhan University, Wuhan, China; ^2^ Department of Radiology, Zhongnan Hospital of Wuhan University, Wuhan University, Wuhan, China

**Keywords:** macrophage polarization, tumor-associated macrophages, tumor microenvironment, immune escape, metabolic reprogramming

## Abstract

Macrophages are one of the most important cells in the innate immune system, they are converted into two distinct subtypes with completely different molecular phenotypes and functional features under different stimuli of the microenvironment: M1 macrophages induced by IFN-γ/lipopolysaccharides(LPS) and M2 macrophages induced by IL-4/IL-10/IL-13. Tumor-associated macrophages (TAMs) differentiate from macrophages through various factors in the tumor microenvironment (TME). TAMs have the phenotype and function of M2 macrophages and are capable of secreting multiple cytokines to promote tumor progression. Both tumor cells and macrophages can meet the energy needs for rapid cell growth and proliferation through metabolic reprogramming, so a comprehensive understanding of pro-tumor and antitumor metabolic switches in TAM is essential to understanding immune escape mechanisms. This paper focuses on the functions of relevant signaling pathways and cytokines during macrophage polarization and metabolic reprogramming, and briefly discusses the effects of different microenvironments and macrophage pathogenicity, in addition to describing the research progress of inhibitory drugs for certain metabolic and polarized signaling pathways.

## Introduction

The latest global data released by the World Health Organization International Cancer Research Agency (IARC) show that the number of new global cancer cases in 2020 rose to 19.3 million, with 10 million deaths. At present, the main treatment methods of cancer are surgical treatment, chemotherapy, radiotherapy, molecular targeted therapy and immune checkpoint inhibitor treatment. Immunotherapy uses immune cells in the tumor microenvironment (TME) to specifically identify and attack cancer cells, which has better application prospects. The TME contains a variety of immune cells, such as macrophages, effector T cells, natural killer cells and dendritic cells. Among these, macrophages are the largest and critical population of innate immune cells in the TME. Macrophages are derived from bone marrow hematopoietic stem cells and embryonic yolk sac tissue. Macrophages involved in the pathogen response are derived from monocyte precursors in the blood circulation; these cellular precursors are found in chemokines such as CCL5, CCL7, CCL20. Then cytokines such as the macrophage colony-stimulating factor and macrophage migration inhibitory factor (MIF) are recruited and infiltrate into tumor tissues, and macrophage inflammatory protein 1α causes, differentiation of macrophages into TAM under the influence of the TME. These cells also secrete a variety of cytokines, chemokines, and proteases and promote tumor cell growth, invasion, metastasis, and drug resistance (1).

TME is the environment surrounding a tumor during its own growth or mutation, and includes cancer cells and invasive immune cells. Tumor cells can promote their own growth by improving this environment, and the body can inhibit or kill tumor cells by changing the environment surrounding the tumor cells (2). The interaction between the metabolic reprogramming of tumors and immune cells is one of the determinants of the tumor immune response (3). Tumor metabolism plays a key role in maintaining tumorigenesis and survival, and also affects immune cells by releasing metabolites. Tumor cells can regulate energy metabolism through metabolic reprogramming to promote rapid cell growth and proliferation, and select appropriate metabolic modes depending on the concentrations of external nutrients and different stress conditions. This complex metabolic pattern also exists in immune cells, and different metabolic patterns also affect the differentiation of different immune cell subsets. Indeed, metabolic competition between tumor and immune cells limits the nutrient supply of immune cells. The metabolites of tumor cells, such as high levels of lactate, low pH, hypoxia and high levels of reactive oxygen species (ROS) are found in the TME and lead to tumor progression and immune escape (4).

Tumor angiogenesis is one of the most important mechanisms of tumor growth and invasion. TAM can be involved in tumor angiogenesis through the secretion of pro-angiogenic factors, including VEGF-A, EGF, TGF-, TNF-, IL-1, and IL-8 (5). Hypoxia is another crucial condition for tumor angiogenesis. Studies have demonstrated that TAM in the hypoxic TME significantly induces tumor angiogenesis, a process accompanied by the polarization and metabolic reprogramming of macrophages (6). Hypoxia inducible factor (HIF) is involved in the aerobic glycolysis process of TAM, while studies have demonstrated that the expression of HIF in TAM is significantly increased and induce the production of VEGF-A (7). This suggests the relevance of tumor angiogenesis and reprogramming of TAM metabolic reprogramming.

Immune cells undergo metabolic reprogramming during proliferation, and differentiation and when performing effector functions, and are essential for the immune response process (8). Cross-talk between macrophages and other innate immune cells in the TME plays an important role in the metabolic reprogramming process of immune cells. Both tumor-associated neutrophils (TANs) and TAM can affect cancer growth and metastasis, and their spatial distribution in the TME is interrelated. Studies have shown that co-cultured TANs and TAM can greatly secrete OSM and IL-11, which promotes the proliferation and invasion of intrahepatic cholangiocarcinoma (ICC) cells. Meanwhile, the team also found that there may be a positive feedback loop between TAN and TAM, TAN expresses CCL2, CCL5 and CSF1 mediate TAM infiltration, while TAM expresses CXCL8 and CSF3 promote TAN infiltration (9). This process is accompanied by the metabolic reprogramming of both TAN and TAM. Thus, understanding how the metabolic reprogramming of tumors and immune cells regulates the antitumor immune response could allow us to identify therapeutic approaches for targeted metabolic pathways in antitumor immunotherapy. This review focuses on the effects of macrophage activation and polarization in the TME on tumor growth and development.

## Metabolism-Related Signaling Pathways in Macrophage Polarization

Macrophage polarization refers to the morphological, functional and phenotypic differentiation of macrophages under the action of different microenvironmental signaling *in vitro* and *in vivo*. According to their immunological functional differences, polarized macrophages can be divided into M1 macrophages in the classical activation pathway and M2 macrophages in the alternative activation pathway (10), while M2 macrophages can in turn be subdivided into M2a (induced by IL-4 or IL-13), M2b (induced by immune complexes in combination with IL-1β or LPS), M2c (induced by IL-10, TGF-β or glucocorticoids) and M2d (induced by TLR + adenosine A2A receptor ligands or IL-6) (11). Different M2 macrophage subtypes can be identified by different surface markers, M2a highly expresses CD206, Arg1, Ym1, FIZZ1 and TGF-β; M2b highly expresses IL-1β, IL-6 and TNF-α; M2c highly expresses CD206 and CD163; M2d highly expressed VEGF and IL-10 (12). M1 macrophages are generated upon stimulation by LPS and IFN-γ. They, produce ROS and induce the production of inducible nitric oxide synthase (iNOS) and a large number of inflammatory factors such as TNF-α, IL-6 and IL-1β, and play important roles in physiopathologic processes such as killing pathogens, resistance to parasite and tumor cells, and anti-inflammatory responses. M2 macrophages, mainly produced by IL-4/IL-10/IL-13 stimulation, can be classified into multiple subtypes based on differences in induced environmental and functional typing, with immunomodulation, immunosuppression and tissue remodeling (13).

The above is a traditional typing method, and with further study of the macrophage polarization process, it was found that the M1 and M2 alone do not well distinguish the macrophage phenotype. Therefore, macrophages were genotyped based on the expression of macrophage surface markers (14). Furthermore, it has also been suggested to incorporate stimulators during naming to differentiate between macrophages (15).

**Figure 1:**
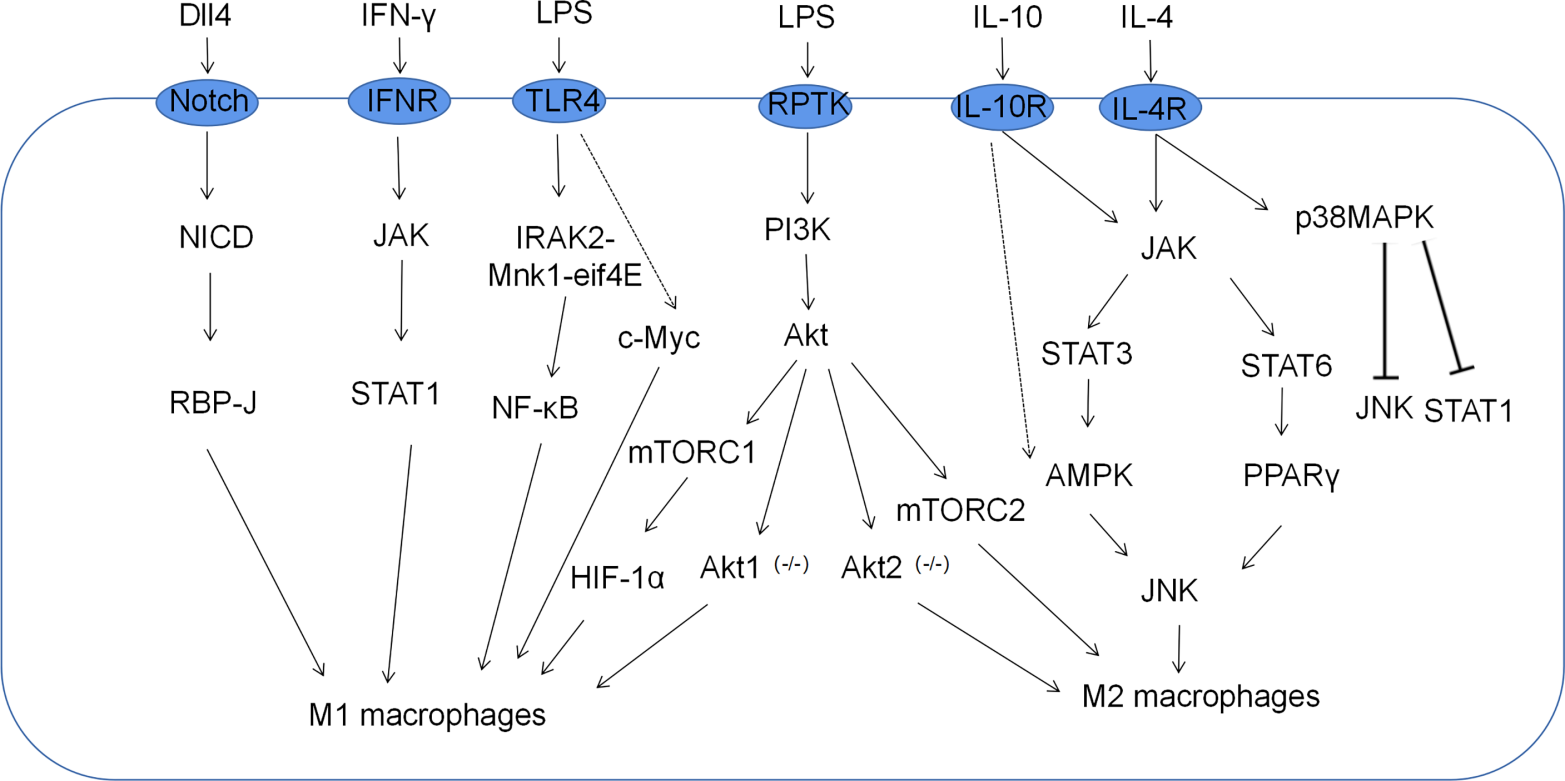
Related signaling pathways for macrophage polarization. M1 macrophages, which are classically activated by IFN-γ and LPS, are mediated by the PI3K-AKT-mTOR-HIF-1 signaling cascade pathway. Moreover, JAK-STAT1, Notch, and NF-κB also play important roles in the polarization of M1 macrophages. M2 macrophages alternatively activated in response to IL-4/IL-10 are mediated by JNK-STAT axes.

**Figure 2:**
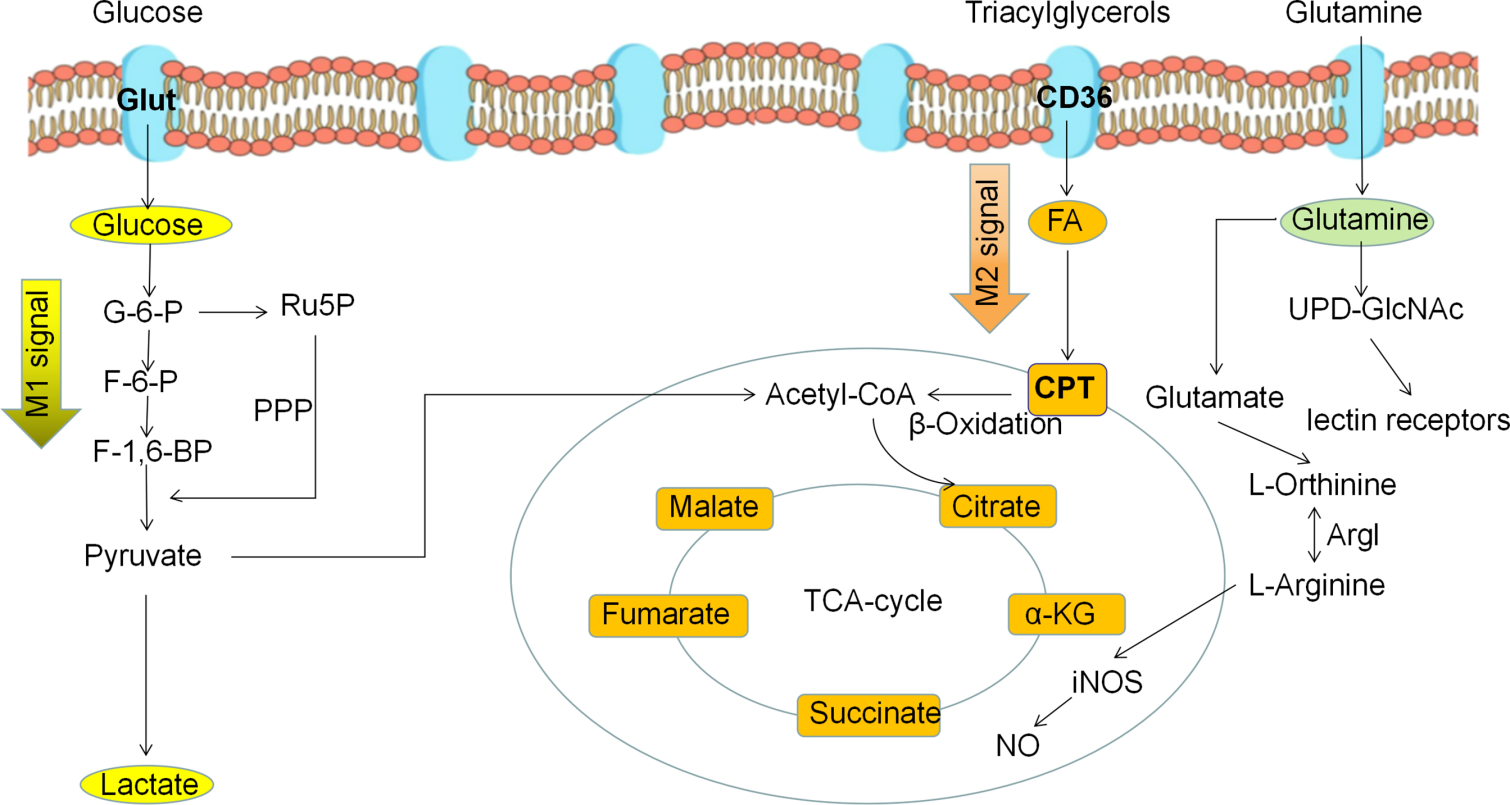
Metabolic reprogramming of polarized macrophages. M1 macrophages mainly use glycolysis as the predominant energy supply mode. M2 macrophages mainly use fatty acid oxidation and oxidative phosphorylation as the main energy supply mode.

Macrophage polarization is a complex process co-regulated by multiple signaling molecules and their signaling pathways. The main signaling pathways are JAK/STAT, PI3K/AKT, JNK and Notch pathways (**Figure 1**) (16). Macrophage metabolism is similarly regulated by a variety of signals and pathways, including the HIF, PI3K/AKT, PPAR and AMPK pathways, some of which play a key role in macrophage polarization, along with the associated cytokines (**Figure 2** and **Table 1**).

**Table 1:** Signaling molecules involved in the metabolic reprogramming of macrophages.

Signaling pathway	Signaling molecules	Effects on macrophage metabolism	Effects on polarization of macrophages
PI3K	AKT, miR-155, NF-κB, Glut1, HK2, PFK2, GPCR	Participate in glycolysis	M1 macrophages (17)
mTORC1	AKT, TSC1, TSC2, AMPK, HIF-1α, FOXK1, SREBPs	Involved in protein synthesis, adipogenesis, glycolysis and inhibit autophagy	M1 macrophages or M2 macrophages (18, 19)
mTORC2	AKT, IRF4, STAT6, PKC, SGK1	Cytoskeletal composition and lipid metabolism	M2 macrophages (18, 20)
HIF -1α	LDHA, PDK, GAPDH, GLUT1, HK2, PKM2, PGK1, VHL, JAB1, PFK	Involved in glycolysis and lactate production	M1 macrophages (21, 22)
HIF-2α	IL-6, IL-1β, Arg1, VEGF	Promote tumor angiogenesis	M2 macrophages (23) (24)
AMPK	IL-10, STAT3, AKT, mTORC1, TGF-β	OXPHOS	M2 macrophages (25)
PPARs	PPARα, PPARβ, PPARγ, PPARδ, STAT6, IL-10, AMPK, Triacylglycerol, CPT, IL-4, PGE2	Lipid metabolism	M2 macrophages (26)
Glutamine	GPT2, α-KG, UDP-GlcNAc	Involved in glutamate metabolism, TCA cycle and UDP-GlcNAc synthesis	M2 macrophages (27)
Lactate	Arg1, mTORC2, ERK, Notch	OXPHOS	M2 macrophages (28)
C/EBPβ	Arg-1, IL-10, IL-13ra, Msr1	Involved in cell differentiation, tumorigenesis and energy metabolism	M2 macrophages (29)
Notch	TLR4, Hes1, NF-κB, Dll4	Involved in aerobic glycolysis	M1 macrophages (30)

### PI3K/AKT Pathway

M1 macrophages mainly utilize glycolysis to meet biosynthesis and energy needs. M1 macrophages are activated by LPS *via* the PI3K/AKT pathway. LPS upregulates NF-κB expression by activation of the PI3K/AKT pathway and induce M1 macrophage polarization. Knockdown of AKT1 was shown to result in negative transcriptional regulation of miR-155, with activation of RelA/NF-κB, inhibiting the cytokine signaling suppressor 1 and ultimately promoting M1 macrophage polarization (31).

Meanwhile, the activated PI3K/AKT pathway can upregulate multiple glycolytic key enzymes and enhance the ability of macrophage to uptake and utilize glucose (32). G-protein coupled receptors (GPCRs), receptor tyrosine kinases (RTKs) and Toll-like/IL-1 receptors (TLR/IL1Rs) all activate the PI3K/AKT pathway, enhance cancer-associated inflammation in TAMs and promote glycolytic progression in M1 macrophages (33). It has been demonstrated that Renalase is a secreted flavin that acts as a survival factor after ischemic and toxic damage, signaling through the plasma calcium channel PMCA4b and activating the PI3K/AKT and MAPK pathways, with significantly increased expression in primary melanoma and CD163(+) tumor-associated macrophages, which in turn regulates the metabolic reprogramming of tumors and TAM (34). Therefore, it can be argued that both macrophage polarization and metabolic reprogramming processes are affected when LPS activates the PI3K/AKT pathway.

### mTOR Pathway

The mammalian rapamycin target (mTOR) is a serine/threonine kinase consisting of two scaffold complexes mTOR complexes 1 (mTORC1) and mTOR complex 2 (mTORC2), located downstream of the PI3K/AKT/mTOR signaling pathway and is a key site in regulating energy supply, biosynthesis, glycolysis and lipid metabolism (35). mTORC1 primarily promotes protein synthesis, adipogenesis, energy metabolism, autophagy inhibition and lysosomal formation processes, while mTORC2 plays a critical role in cytoskeletal composition, cell survival and metabolism (20).

mTORC1 can regulate the polarization of M1 macrophages as well as the metabolic reprogramming processes. In M1 macrophages, the mTORC1/HIF-1α axis is indispensable for the transcription of pro-inflammatory cytokines and metabolic genes related to glycolysis (36). It was demonstrated that mTORC1 affects glycolysis, the pentose phosphate pathway, and lipid metabolism processes by activating the gene transcription of hypoxia-inducible factor and sterol regulatory element-binding protein (37). Furthermore, FOXK1 directly regulates mTORC1 signaling and CCL2 expression in a manner independent of NF-κB, promoting tumor progression through the secretion of CCL2 (38). M2 activated macrophages use fatty acid oxidation and oxidative phosphorylation (OXPHOS) as the main metabolic pathways while increasing glucose utilization, in which mTORC2 acts in parallel to the IL-4Rα/STAT6 pathway to promote increased glycolysis during M2 activation by inducing the transcription factor IRF4 (39). Some studies have established that constitutive activation of mTORC2 can promote the polarization of M2 macrophages in TAMS, which in turn leads to the immunosuppression of TME. However, existing studies demonstrate that IL-4 signaling selects the Akt-mTORC1 pathway to regulate ATP citrate lyase, leading in increased histone acetylation and M2 gene induction (19). This suggests that mTOR, an energy regulatory center, may have a more complex role in the process of macrophage polarization. Thus, mTORC1 and mTORC2, as key nodes in macrophage polarization and metabolism, receive the effects of multiple signaling molecules in the TME and are not only involved in M1 or M2 typing, but serve as nodes in the interconversion of M1 and M2 typing.

The tuberous sclerosis complex (TSC1 and TSC2) are negative regulators of mTOR activity, and the main role is to inhibit mTORC1 signaling. On the PI3K/AKT/TSC/mTORC1 signaling pathway, upstream signaling molecules inhibit the TSC complex *via* the PI3K-AKT pathway to activate mTORC1 kinase activity (40). Molecular studies show that TSC1 can inhibit M1 macrophage polarization by inhibiting RasGTPase/Raf1/MEK-ERK signaling, whereas TSC1 promotes M2 macrophage polarization through the mTOR-dependent CCAAT/enhancer-binding protein-β pathway (41). This suggests a critical role of TSC in coordinating macrophage polarization through mTOR-dependent and independent pathways.

### HIF

HIF is a heterologous protein dimer composed of a α subunit and a β subunit that can regulate a range of gene expression of cells in a hypoxic environment. HIF is divided into three subtypes, HIF-1α, HIF-2α and HIF-3α, in which the role and function of HIF-1α and HIF-2α have been extensively studied.

In inflammation and TME, macrophages undergo metabolic reprogramming to be adapted to hypoxic conditions, involving many HIF-1α -dependent gene expression, with many regulatory processes involved (21). PI3K/AKT/mTOR, RAS/RAF/MEK/ERK and IKK/NF-kB are upstream regulatory signals of HIF-1a and can induce increased HIF-1a expression (22). HIF-1a is constitutively expressed. Normally, HIF-1α can be ubiquitinated by VHL for rapid degradation, maintaining the normal function of the body. However, under hypoxia, HIF-1a is required for glycolytic gene expression, including those encoding the GLUT1, LDH-A, hexokinase, phosphofructokinase, pyruvate kinase, and GAPDH. During glycolysis, HIF-1α is considered a key factor in determining how cells convert pyruvate into lactate. In normal cells, the pyruvate produced by glycolysis enters the tricarboxylic acid cycle for oxidative phosphorylation (OXPHOS). However, in tumors, as well as in some immune cells, HIF-1α can facilitate the aerobic glycolysis by conversion of pyruvate to lactase by promoting the expression of lactic acid dehydrogenase (LDHA) and pyruvate dehydrogenase kinase (PDK) (42, 43). Furthermore, the glycolytic process mediated by AKT/mTOR/HIF-1α was shown to be associated with training immunity in monocytes and macrophages, and the researchers found that HIF-1a-deficient mice were unable to produce a trained immune response to bacterial sepsis (44). HIF-1α induced by pro-inflammatory cytokines is strongly associated with M1 macrophages. Overexpression of HIF-1α induces M1 macrophage polarization *via* NF-κB and upregulates genes related to glycolytic metabolism (45). In addition, it has been demonstrated that HIF-1α-stabilizing long noncoding RNA (HISLA) blocks PHD2 and HIF-1α interaction and thereby suppresses HIF-1α hydroxylation and degradation, while HIF-1α promotes aerobic glycolytic processes in tumor cells and released lactate upregulates HISLA, in macrophages that constitutes a feedforward loop between TAMs and tumor cells (46).

Among the known functions, HIF-1α and HIF-2α have partially overlapping roles. However, HIF-2α expression is more restricted for structural reasons. Studies indicated that HIF-1 α and HIF-2α are expressed in M1 and M2 macrophages, respectively (24). LPS or IFN-γ significantly increased HIF-1α protein abundance and inhibited HIF-2α gene expression. In contrast, IL-4 or IL-13 significantly increased the HIF-2α protein abundance. Meanwhile, HIF-2α induces Arg1 gene expression, a specific marker of M2 macrophages (47). HIF-1α and HIF-2α have been thought in the past to be involved in the polarization process of M1 macrophages and M2 macrophages, respectively. However, a recent study noted that in Clear cell renal cell carcinoma, HIF-1α highly expressed in TAMs is associated with poor prognosis and polarization of M2 macrophages, and HIF-2α with good prognosis, in contrast to previous studies (48). This suggests that the functions of HIF-1α and HIF-2α are plastic during tumor progression, play an important regulatory role in the metabolic reprogramming of tumor cells and macrophages, and produce interesting changes under the influence of TME.

### AMPK

Adenosine 5′-monophosphate-activated protein kinase (AMPK) is an energy sensor that regulates energy homeostasis in response to metabolic stress (49). Anti-inflammatory cytokines such as IL-4 and TGF-β have been shown to promote M2 macrophage polarization and to favor glucose metabolism *via* OXPHOS (50). AMPK is a key regulator of OXPHOS. It is activated by adenosine, its substrate and anti-inflammatory factors, and puts macrophages into an immunosuppressed state. Activated AMPK will induce macrophages to take OXPHOS as the main metabolic mode and promote the polarization of M2 like macrophages (51). Conversely, LPS-induced M1 macrophages show inhibition of AMPK and favor glycolysis as the major glucose metabolic pathway. Some results suggest that AMPK is involved in the polarization and metabolic reprogramming of M2 macrophages. AMPK plays a key role in the M2c macrophage activation pathway induced by IL-10. AMPK can inhibit pro-inflammatory responses and promote M2 macrophage polarization by negatively regulating LPS-induced IkappaB-degradation and positively regulating Akt activation (52). Follow-up studies by this research team demonstrated that AMPK promotes the IL-10-mediated macrophage polarization to the M2 phenotype through the PI3K/Akt/mTORC1 and STAT3 signaling pathways (25).

### PPARs

The peroxisome proliferator activation receptor (PPAR) is a key sensor for lipid metabolism. As a nuclear receptor and transcription factor, PPARs can directly initiate or inhibit the expression of many target genes and play regulatory roles in cellular glycolipid metabolism. IL-13 and IL-4 secreted by adipocytes or Th2 cells activate STAT6 and phosphorylate AMPK, resulting in increased expression of PPAR-δ with ACE, inhibiting M1 polarization and promoting the expression of type M2 genes (53, 54). PPARγ depletion results in the inhibition of M2 macrophage polarization. Studies have shown that M2 macrophage polarization was inhibited by arachidonic acid, but was inversely promoted by its derived metabolite prostaglandin E2 (PGE2). PPARγ connects the two processes *via* OXPHOS. PGE2 enhanced OXPHOS through inhibiting PPARγ, resulting in the alternative activation of macrophages (55). PPARδ, a member of the PPAR family, plays an important role in the clearance of apoptotic cells and is involved in tumor construction. Studies have shown that PPARα/β promotes TAM activation through enhanced IL-10 expression and induces the polarization process in M2 macrophages (56).

### Other Signaling Pathways

There are many other signaling molecules involved in the polarization and metabolic processes of macrophages. The transcription factor c-Myc is involved in glycolysis and glutaminolysis of immune cells. It was demonstrated that c-Myc plays an important role in the induced macrophage polarization and metabolic reprogramming process by LPS. The c-Myc is required to increase early glycolysis and regulates the pro-inflammatory and microbial-killing functions of inflammatory macrophages (57). FOXO1 promotes the transcriptional polarization of M2 macrophages and the recruitment of M2 macrophages at TME through the transcriptional modulation of CCL20 and CSF-1 (58). The C/EBPβ is a member of the C/EBP family. It has been demonstrated that the CREB-C/EBPβ cascade can induce M2 macrophage-specific gene expression, including Arg-1, IL-10, IL-13ra, and Msr1 (29). The JAK-STAT1 pathway mediated by IFN-γis a putative pathway for M1-like macrophage polarization (59). Currently, most consider JAK-STAT1 as a key node in macrophage polarization, but further studies are needed to determine whether it regulates the metabolic processes of macrophages. Moreover, DII4-Notch is also an important pathway for the polarization of M1-like macrophages. One report showed that the lactate metabolism regulated by activated Notch signaling may be involved in MDSC differentiation and TAM maturation, so we can speculate that the Notch pathway may be involved in macrophage metabolic reprogramming by regulating lactate metabolism (30).

In addition, epigenetic regulation has important implications for macrophage polarization and metabolism. Epigenetic regulation mainly consists of three aspects, including DNA methylation, non-coding RNA and histone modifications. It has been reported that JMJD3 contributes to decreased H3K27 methylation levels and increases the transcriptional activity of M2 marker genes (60). Meanwhile, it was found that activated PERK promotes serine biosynthesis through the downstream transcription factor ATF-4, which leads to enhanced mitochondrial function and α-ketoglutarate production required for JMJD3-dependent epigenetic modification, thus promoting mitochondrial respiration and lipid oxidation in M2 macrophages (61). Furthermore, it has been demonstrated that the transcription factor CTCF recruits histone acetyltransferase E1A binding protein p300 to promoter regions by binding to downstream acting targets, thereby enhancing histone acetylation and gene transcription and promoting M2 polarization of TAM (62).

In summary, polarization and metabolic reprogramming of macrophages are the result of coregulation by multiple signaling pathway interactions that produce adaptive changes based on differences in the microenvironment.

## Effects of The Microenvironment on Macrophage Polarization and Metabolism

**Figure 3:**
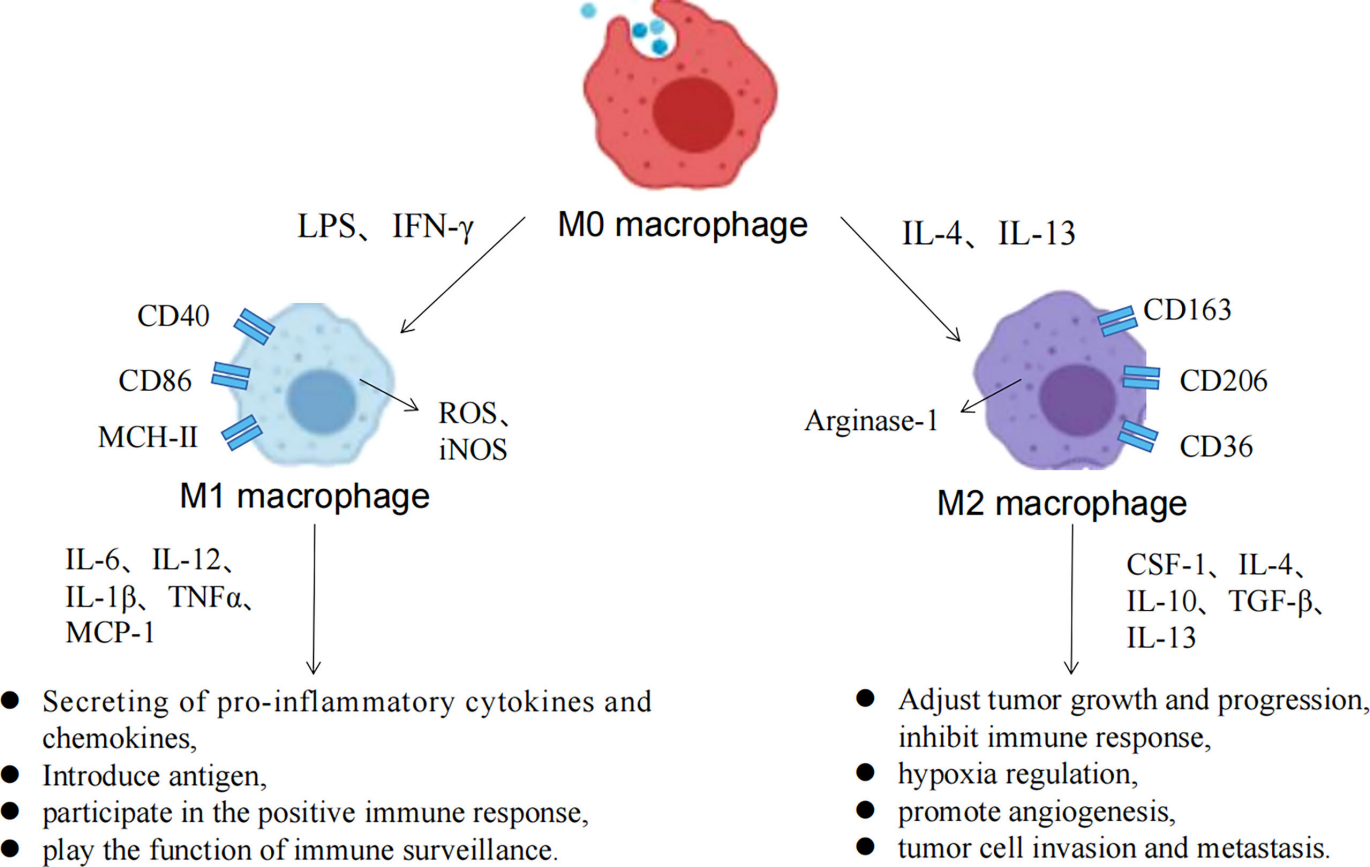
Macrophage polarization process and the associated functions of M1/M2 macrophages.

Macrophages have strong plasticity, and can be polarized into different subtypes by the TME, with a two-sided relationship with tumors (**Figure 3**) (63). M1 macrophages can be activated by IFN-γ and TLRs to recognize tumor cells through cell surface antigens, releasing tumor killer factors such as NO and ROS, which have antitumor effects. M2 macrophages can be activated by IL-4 and IL-13 and are regulated by multiple transcription factors and secreted cytokines when regulating tumor growth progression, which inhibits immune response, hypoxia regulation, angiogenesis, invasion, and metastasis. Metabolic regulation between TME and TAMs is gaining attention, and all components of the TME rely on nutrients for survival, maintenance, and proliferation (64). At the same time, competition and symbiosis between tumor cells and other components of the TME can influence each other, and an excess of metabolites can lead to the reprogramming of immune cells, resulting in new phenotypes and functions. Normally, immune cells in the microenvironment are in a relative resting state and which usually metabolize glucose to pyruvate within the mitochondrial TCA cycle to acetyl-CoA or undergo fatty acid oxidation. However, when the body is stimulated by infection, tumors and other factors, immune cells such as macrophages need to undergo metabolic reprogramming to change the cellular metabolic pathways to obtain large amounts of energy and metabolic intermediates to meet their biosynthetic needs, to undergo proliferation and differentiation and perform effector functions (65).

**Figure 4:**
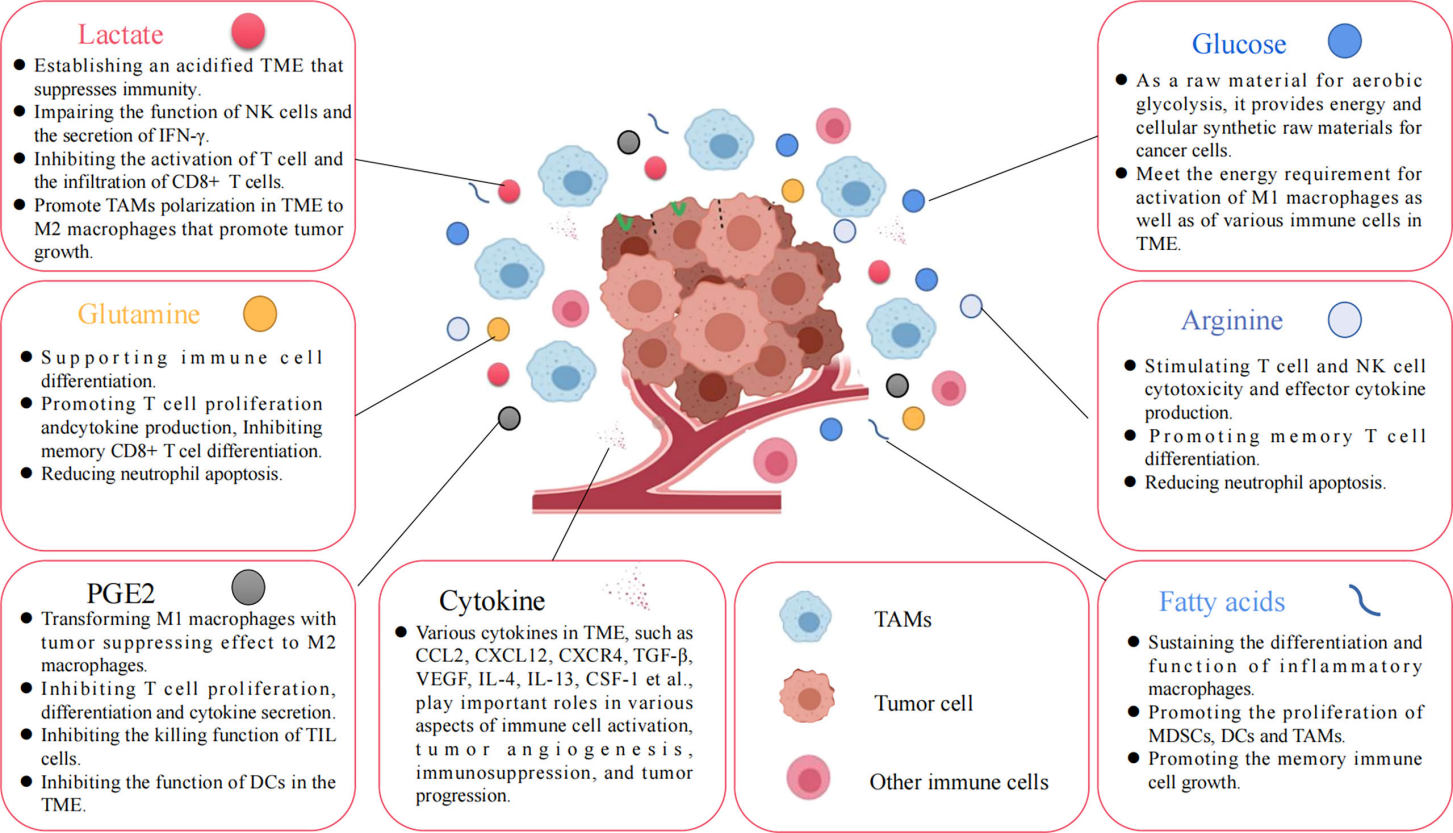
Various nutrients and metabolites in the tumor microenvironment. Glucose, amino acids, glutamine, fatty acids, and other metabolites, or growth factors in the TME are all important factors affecting tumor cell proliferation and immune cell function.

Under normal physiological conditions, macrophages use OXPHOS as their primary metabolic pathway for their energy requirements. However, macrophages undergo polarization and metabolic reprogramming when stimulated by external pathogens, cytokines, and tumor metabolism, such as LPS, IFN-γ, TNF-α, IL-1, IL-4, IL-10. M1 macrophage metabolism changes from glucose OXPHOS to glycolysis, and is accompanied by increased lactate release, decreased oxygen consumption rates, and glutaminolysis (66). The aforementioned changes in glucose metabolism patterns can produce the metabolic intermediates and meet the energy needs of the M1 macrophages. In addition, disruption of the TCA cycle in M1 macrophages causes the accumulation of succinate and citrate, which stabilize HIF-1α and IL-1β by inhibiting proline hydroxylase activity, to further accelerate glycolytic metabolism and drive inflammatory responses; citrate participates as a synthetic substrate for NO and prostaglandin (67). Unlike the metabolic pattern of M1 macrophages, M2 macrophages have a complete TCA cycle and a significant increase in oxygen consumption upon activation and mainly rely on OXPHOS and FAO for metabolic energy supply. Moreover, the polarization of M2 macrophages also depends on glutamine, which can supplement TCA cycle intermediates for the synthesis of biomolecules such as lipids, and provide a nitrogen source for the synthesis of non-essential amino acids and nucleic acids. Thus, altered glycolipid metabolism in macrophages determines M1/M2 polarization and regulates its immune function (**Figure 4**). Metabolic reprogramming of macrophages is mainly caused by changes in the TME, and polarized macrophages are not unchanged. M1 macrophages and M2 macrophages can be mutually transformed through action of lactate as well as some cytokines (28).

### The Influence of Glucose-Rich Milieu on Macrophage Function

The main energy source for the normal function of macrophages is glucose. Glucose is metabolized within the cell through three main pathways: glycolysis, the pentose phosphate pathway (PPP) and the TCA cycle. The main steps of glycolysis are completed in the cytoplasm, where glucose is decomposes into pyruvate in an aerobic environment. It then reenters the mitochondria to participate in the TCA; which decomposes glucose into lactate in an anaerobic environment and produces ATP (68). The pyruvate produced by normal glycolysis further produces more ATP through OXPHOS. However, in tumor cells in an aerobic environment, glycolysis does not enter the TCA, which causes high consumption of glucose in the TME and produces metabolites such as lactate, which affect the metabolic reprogramming of immune cells in TME (69). Glycolysis powers macrophages and other cells more rapidly than OXPHOS while providing cells with essential intermediate metabolites. Normally, naive M0 macrophages acquire energy through the efficient process of OXPHOS. However, polarized macrophages choose appropriate metabolic patterns based on the TME and their own energy metabolism characteristics.

TAMs constitute the largest immune cell population in the TME and play an immunosuppressive role during tumor development. Since cancer cell energy metabolism is mainly dependent on glucose, they consume large amounts of glucose in the TME and undergo aerobic glycolysis to meet the rapidly growing tumor energy needs. Thus, TAM metabolic features shift to OXPHOS and FAO metabolism and exhibit a function similar to M2 macrophages in a low glucose TME and demonstrate an immunosuppressive effect (70, 71). Minor differences in environmental stimuli can give rise to significantly different macrophage phenotypes and metabolic profiles. Macrophages can exhibit different reactivity even with the same stimulus. TAMs actually have a higher glucose uptake capacity and high levels of glycolytic metabolism similar to M1 macrophages to support their cytokine profile and function. Proteomic analysis revealed that glycosylase expression including HK2 was upregulated in macrophages stimulated with tumor extracts from breast cancer patients (72), which is consistent with pancreatic ductal adenocarcinoma (PDAC) (73) and non-medullary thyroid carcinoma (74). At the same time, lactate release of glycolysis into the TME in cancer cells also upregulated HIF-1a expression in TAMs, resulting in increased glycolysis and the M2-like state (75, 76). In addition, *in vivo*, macrophages are able to repolarize from the M2 to the M1 phenotype, and can co-express M1 and M2 polarization features after tumor progression (77). It has been shown that a novel subtype of CD19+ TAMs was found in HCC, and the results showed that glycolysis may be an innate feature benefiting tumor progression. Glucose-rich milieu may induce a macrophage polarization shift to a M2-like phenotype. It was found that O-GlcNAc in TAMs can enhance glucose flow to promote polarized M2 macrophages through the hexosamine biosynthetic pathway (HBP) and drive cancer progression and immune evasion (78). Indeed, compared to immune cells, tumor cells rely more on glucose to support their growth than TAMs, and this nutritional competition between tumor and immune cells is clearly unfavorable for tumor cell proliferation. In conclusion, the glucose-rich environment favors macrophage polarization to the M1 isoform and plays the tumor-suppressive role in M1 macrophages.

### The Influence of Free Fatty Acid-Rich Milieu on Macrophage Function

Under normal conditions, the expression of key enzymes of fatty acid anabolism in tumor cells, such as sterol regulatory element binding protein and fatty acid synthase (FAS) is increased, and the anabolism of fatty acids dominates. Fatty acid metabolites released into the TME can use multiple pathways to affect the metabolic phenotype and function of immune cells. The effect of tumor fatty acid metabolites on macrophages in different polarized states varies significantly. Among them, M1 macrophages, after uptake of excessive amounts of unsaturated fatty acids, can stimulate the production of IL-1α, leading to increased inflammation, which will further stimulate increased fatty acid synthesis in macrophages and contribute to the inflammatory function of M1 macrophages; M2 macrophages, in contrast, take up triglycerides in the microenvironment mainly through the fatty acid receptor CD36, resolved by lysosomal acid lipase, through high levels carnitine palmitoyl transferase 1α(CPT1), which mediate the conversion of fatty acid mitochondria transmembrane, coordinating mitochondrial respiration and fatty acid oxidation, while reducing the production of inflammatory cytokines and increasing fatty acid metabolism (79).

M1 macrophages are primarily dependent on glycolytic metabolism; however, fatty acid levels in cells can also significantly affect cell function. Research has shown that the performance of the inflammatory function in M1 macrophages requires the participation of the fatty acid synthesis pathway (80). Inflammatory stimuli such as LPS and cytokines trigger increased fatty acid synthesis in M1 macrophages. When macrophage colony-stimulating factor (M-CSF) induces macrophage differentiation, the sterol regulation factor binding to the transcription factor 1c is upregulated, with increased expression of fatty acid synthesis-related target genes and increased lipid synthesis. Alternatively, mitochondrial uncoupled protein 2 promotes NLRP3 inflammatory body activation by regulating FASN and stimulating fatty acid synthesis, exacerbating the inflammatory response to during sepsis (81). In brief, these studies suggest that fatty acid synthesis is required for the differentiation and function of inflammatory macrophages.

The main mechanisms by which M2 macrophages exert immunosuppressive effects include the expression and functional activity of arginase and the S-nitrosylation of surface proteins in infiltrating T cells (including T cell receptors). The metabolic characteristics of M2 macrophages are clearly distinct from M1 macrophages, and the process driving the M1 macrophage glycolytic switch is downregulated in M2 macrophages and exhibits higher levels of FAO, mitochondrial quality and mitochondrial respiration rate, and decreased OXPHOS significantly inhibited the M2 phenotype, including anti-inflammatory cytokine secretion and expression of M2 activation markers. CPT1, which is located in the outer mitochondrial membrane and is capable of transporting fatty acids through the mitochondrial membrane, is a rate-limiting step in β-oxidation. Upregulation of CPT1α expression in macrophage cell lines promotes FAO and reduces inflammatory cytokine production (82). This effect is mediated by signaling transduction and transcriptional activator 6 and the peroxisome proliferator activating receptor coactivator 1 β (83). However, it has been shown that CPT2 deletion does not affect M2 macrophage polarization, although it inhibits FAO (84). Therefore, CPT1 has additional roles other than fatty acid transport during M2 polarization and a complex mechanism of action. One study showed that M2 takes up triacyl glycerol substrate through the scavenger receptor CD36, which is followed by adipolysis by lysosomal acidic lipase, and this is the first finding that cellular intrinsic lysosomal lipolysis plays a critical role in M2 activation (85).

## Control of Cellular Metabolism as a Target for Cancer Therapy

Given that TAMs promote tumor development, there are two major strategies for targeting them, reducing the number of TAMs or controlling metabolic reprogramming. Metabolic alterations after macrophage polarization are the major driver in mediating macrophage function. Furthermore, the metabolic requirements of tumor cells are significantly increased compared to normal differentiated cells, and cancer cells exhibit extremely high metabolic activity such as aerobic glycolysis and glutamine metabolism (86). Therefore, the development of therapeutic methods for key metabolic enzymes and metabolic pathways may have important clinical significance (**Table 2**) (101).

**Table 2:** Current status of various metabolic pathway inhibitors.

Inhibition of pathways	Inhibitor	Research status
Inhibits hexose kinase	2-DG	Phase I clinical trial [87]
Inhibits PKM2	Shikonin	*In vitro* experiments [88]
	HA344	*In vitro* experiments [89]
Inhibits PDK	DCA	Phase II clinical trial [90]
Inhibits HIF-1α	PX-478	Phase I clinical trial [91]
	EZN-2968	Phase I clinical trial [92]
Inhibits HIF-2α	Belzutifan	Phase II clinical trial [93]
Inhibits PI3K	CYH33	Phase I clinical trial [94]
Inhibits AKT	MK-2206	Phase II clinical trial [95]
Inhibits mTOR	Temsirolimus	Phase II clinical trial [96]
	Everolimus	Phase II clinical trial [97]
	Ridaforolimus	Phase I clinical trial [98]
	Sapanisertib	Phase II clinical trial [99]
	AZD8055	Phase I clinical trial [100]

### Glycolysis Inhibition

2-Deoxy-D-glucose(2-DG), a non-metabolizable glucose analogue, suppresses glycolysis by acting on hexose kinase (102). A completed phase I clinical trial evaluated the effect of 2DG alone and in combination with docetaxel on advanced solid tumors (87). Studies have also shown that low doses of 2-DG inhibition of glucose metabolism combined with a MEK inhibitor induces apoptosis in krasg12d-driven pancreatic ductal adenocarcinoma cells, and experiments also show that this combination treatment inhibited the growth of xenograft pancreatic ductal adenocarcinoma and prolonged total survival (103).

The pyruvate kinase M2 (PKM2) is a key enzyme in the last step of glycolysis, and is also a regulatory site of many signaling pathways, promoting aerobic glycolysis in cancer cells during tumor progression (104). Shikonin was reported as an inhibitor of PKM2 that suppresses cancer cell proliferation and overcomes chemotherapeutic drug-mediated resistance (88, 105). In addition, it has been proposed that HA344 inhibits the final and rate-limiting steps of glycolysis by covalently binding to PKM2 while blocking the effect of inosine monophosphate dehydrogenase activity, and the authors described the considerable potential of HA344 in overcoming cancer resistance (89).

### PDK Inhibition

Dichloroacetate (DCA), a PDK inhibitor that inhibits the Na^+^-K^+^ -2Cl-cotransporter and mitochondrial potassium channel axis, increases reactive oxygen species generation, causes apoptosis in cancer cells, and inhibits tumor growth (106).. DCA has shown good results in clinical trials of head and neck cancer, glioblastoma, and other recurrent brain cancers. Furthermore, it has been demonstrated that DCA-treated melanoma cell line metabolism shows reduced glucose consumption and lactate production, downregulated proliferation, increased apoptosis, decreased activation of the mTOR pathway. Therefore, DCA alone or in combination with mTOR inhibitors has the potential to treat cutaneous melanoma (107).

### HIF Inhibition

HIF-1α is a transcription factor that regulates metabolism, affecting the gene expression involved in glycolysis, angiogenesis and cell proliferation (108). PX-478 acting as a HIF1α inhibitor has antitumor activity against a variety of cancer cell lines under constant hypoxia *in vitro* and *in vivo (* 109). However, in recent years, no additional clinical studies on PX-478 have been reported, and more studies on PX-478 in combination with other drugs are needed to reduce the side effects of the drug itself and improve efficacy. The combination of DCA and PX-478 demonstrated synergistic effects in a variety of cancers, inhibiting cancer proliferation through the production of ROS and apoptosis (110). Currently, phase I clinical trials are ongoing in patients with advanced solid tumors. EZN-2968, an antisense oligonucleotide inhibitor of HIF-1α, has proved potentially beneficial for HCC patients in a phase I clinical study (92). In addition, other drug types, such as inhibitors of microtubule dynamics, Na+/K+ ATPase, translational regulation of HIF-1α by ATR and inhibitors of stabilization of the HIF-1α protein, have been reported in the literature (111).

Belzutifan, an oral HIF-2α inhibitor for treating adults with cancers linked to the rare genetic disorder von Hippel-Lindau disease, has now been approved for clinical treatment (112).

### Inhibition of the PI3K-AKT-mTOR Pathway

Currently, the efficacy of immunotherapy in multiple tumors is less than satisfactory, and the loss of PTEN or activation of the PI3K pathway in cancer cells enhances the resistance to immunotherapy (113). Studies have shown that PI3K-AKT-mTOR overactivation may be an important reason for USP12 downregulation, which leads to increased TAM abundance in the TME and improves tumor resistance to immunotherapy (114). Thus, targeting the USP12-PPM1B cascade may perturb the TME and increase the efficacy of immune checkpoint blockade in certain cancers.

PI3K is an upstream regulatory target of AKT and mTOR and plays an important role in the PI3K-AKT-mTOR signaling pathway. CYH33, a PI3K inhibitor, has been shown to enhance infiltration and activation of CD8T and CD4T cells while attenuated M2 macrophages and regulatory CD4T cells, in combination with the FA synthase inhibitor C75, inhibit tumor growth and enhance host immunity (94).

AKT activates the mTOR signaling pathway to regulate metabolism. Previous studies have shown the limited efficacy of AKT inhibitors as monotherapy in a clinical trial, but combinations with other drugs showed good results (115). Clinical studies have shown that the application of the AKT inhibitor MK-2206 in breast cancer patients can significantly improve the immune characteristics of TME, and can provide the basis for AKT inhibition combined with immunotherapy (116).

mTOR can induce glycolysis, stimulate cell growth, and play a major role in macrophage polarization and metabolic reprogramming, and dysregulation of mTOR expression underlies multiple human diseases. Therefore, targeting mTOR has great potential in tumor therapy (117). Temsirolimus, a specific inhibitor of mTOR, has shown positive effects in patients in several clinical trials (96). It has also been demonstrated that temsirolimus in combination with capecitabine is effective in patients with advanced solid tumors (118). Everolimus is another mTOR inhibitor that shows anti-antitumor efficacy as a single drug or in combination with other drugs in various clinical trials (119) and performs well in a variety of tumors such as advanced breast (97) and neuroendocrine tumors (120). It has entered phase 2 clinical trials. Another mTOR inhibitor, ridaforolimus, showed antitumor activity in hematological malignancies (121), endometrial carcinoma (122) and sarcoma (123). In addition, relevant clinical trials with several other inhibitors such as sapanisertib have been reported and are performing well in phase I clinical trials in renal, endometrial and bladder cancer (124). Results of phase I clinical trials assessing the safety and resistance of AZD8055 to patients with advanced solid tumors are acceptable (100).

Accumulating evidence suggests that epigenetic modifications have the potential for therapeutic development. Analysis of metabolomics in trained monocytes demonstrated that fumarate accumulation promotes the training of immune reprogramming by inhibiting KDM5 histone demethylases, increasing H3K4me3 and H3K27 acetylation, and inducing epigenetic reprogramming of monocytes (125). It has been found that the mir-144/mir-451a cluster induces M1 like repolarization of TAMs by targeting HGF and MIF. In a regulatory circuit, mir-144 can target histone H3K27 methylation catalyzed by EZH2 and EZH2 to silence the mir-144/mir-451a cluster in HCC (126). Thus, demethylation agents may have the potential to treat inflammatory diseases. However, one challenge is that the same pathway used in tumor cells and other cells in TME cells may play the opposite role depending on different cellular environments. It has been shown that mTOR activation has an antitumor effect in hypoxic TAMs but a tumor-promoting effect in cancer cells. The antitumor effect of mTOR inhibitors is mainly achieved by inhibiting this pathway in cancer cells, but the cancer suppressor effect will be offset by drug harmful results on TAMs (127). In this case, mTOR’s drug inhibition in combination with the TAM depletion strategy showed enhanced effects. In addition, the development of novel nanoagents that target and influence TAMs may be another promising alternative for the successful treatment of tumor (128). It has been reported that nanoagents have been developed and tested in clinical trials in primary breast cancer, bone metastatic breast cancer, and glioblastoma (129, 130).

## Conclusion and Perspectives

TAMs are an important part of the TME and immune ecology and have an important role in regulating tumor progression and metastasis. Molecular targeted therapy that regulates TAM metabolism has become a hotspot in research and development. TAM has a high degree of plasticity, and the effects of M1 and M2 macrophages are completely different for tumors. According to the results of current clinical trials, anti-TAM treatment should be combined with traditional chemotherapy to inhibit tumor cells and regulate the TME, which can achieve an obvious treatment effect and improve the treatment effect of patients. However, the current therapeutic strategies related to TAM pay more attention to exhaust TAM in TME and the research of antimetabolizing drugs tends to inhibit tumor cell metabolism rather than TAM.

The mechanisms of the signaling pathways associated to TAMs polarization and metabolism require further investigation. HIF is the most important regulatory signal for metabolism and polarization. However, it is far from clear about how HIF regulates M1/M2 macrophage expression-associated markers. Furthermore, c-Myc has been shown to influence the expression of genes associated with M2 macrophage polarization induced by IL-4, but the connection between c-Myc and metabolism in M2 macrophage polarization remains unclear. In conclusion, the metabolism and polarization of TAMs are highly correlated with tumor progression and treatment. Further study of the molecular mechanism of TAM metabolic reprogramming can lay a solid theoretical foundation for the targeted inhibition of TAM, to enhance the body’s immune response and develop new therapeutic programs.

## Author Contributions

SW drafted the manuscript. GL and YL review and edit the manuscript. YP conceived of the study, and review and edit the manuscript. All authors read and approved the final manuscript.

## Funding

This work was supported by the National Natural Science Foundation of China (81872200, 31900558), the Hubei Provincial Youth Talents Program for Public Health (WSJKRC2022013), Wuhan Young and middle-aged medical backbone talents Training Project (WHQG201904), the Yellow Crane Talent Program of Wuhan City (HHYC2019002), the Natural Science Foundation of Hubei Province (2020CFB298), the Zhongnan Hospital of Wuhan University Science, Technology and Innovation Seed Fund (ZNPY2018090, ZNPY2019002).

## Conflict of Interest

The authors declare that the research was conducted in the absence of any commercial or financial relationships that could be construed as a potential conflict of interest.

## Publisher’s Note

All claims expressed in this article are solely those of the authors and do not necessarily represent those of their affiliated organizations, or those of the publisher, the editors and the reviewers. Any product that may be evaluated in this article, or claim that may be made by its manufacturer, is not guaranteed or endorsed by the publisher.
